# Revisiting IGF2BP1 in Endometrial Cancer: Ethnic-Specific Prognostic Implications and Association with Caspase-3 and pSTAT3

**DOI:** 10.7150/jca.119549

**Published:** 2025-09-29

**Authors:** Hua Ho, Ching-Yu Shih, Chiao-Yin Cheng, Yao-Jen Liang, Yen-Lin Chen

**Affiliations:** 1 Department of Emergency Medicine, Far Eastern Memorial Hospital, New Taipei, Taiwan.; 2 Center for Precision Medicine and Genomics, Tri-Service General Hospital, National Defense Medical Center, Taipei, Taiwan.; 3 Graduate Institute of Applied Science and Engineering, Department and Institute of Life Science, Fu-Jen Catholic University, New Taipei, Taiwan.; 4 Center for Precision Medicine and Genomics, Department of Pathology, Tri-Service General Hospital, National Defense Medical Center, Taipei, Taiwan.

**Keywords:** IGF2BP1, Endometrial cancer, Caspase-3, STAT3, Prognosis

## Abstract

**Background:** Insulin-like growth factor 2 mRNA-binding protein 1 (IGF2BP1) has emerged as a key N6-methyladenosine reader protein involved in RNA stability and oncogenesis across various cancers. Previous studies, primarily based on Western cohorts, have associated high IGF2BP1 expression with poor prognosis and aggressive tumor behavior in endometrial cancer (EC). However, the prognostic significance of IGF2BP1 in East Asian populations, including those from Taiwan, remains unclear.

**Methods:** This retrospective study analyzed 75 paraffin-embedded EC tissue samples from treatment-naïve Taiwanese patients. Immunohistochemical staining was performed to assess IGF2BP1 expression. Patients were categorized into high- and low-expression groups based on a receiver operating characteristic-derived cutoff score of 10. Kaplan-Meier survival analysis and log-rank tests were used to evaluate survival outcomes. Univariable and multivariable logistic regression analyses were employed to explore associations with clinicopathological features and key biomarkers, including caspase-3 and phosphorylated signal transducer and activator of transcription 3 (pSTAT3).

**Results:** Contrary to prior literature, patients with high IGF2BP1 expression (score >10) exhibited significantly better overall survival compared to the low-expression group (log-rank p = 0.029). The high-expression group-maintained survival probabilities above 85% throughout the 4-year follow-up period, while the low-expression group declined below 40%. Furthermore, high IGF2BP1 expression was positively correlated with caspase-3, a key pro-apoptotic marker, and negatively correlated with pSTAT3, a well-known inflammatory and oncogenic signal transducer. These findings suggest that IGF2BP1 may exert context-dependent biological functions in EC, potentially promoting apoptosis and dampening tumor-promoting inflammation in Taiwanese patients.

**Conclusion:** This study provides novel evidence that high IGF2BP1 expression is associated with improved prognosis in Taiwanese patients with EC. These findings highlight potential ethnic differences in IGF2BP1-mediated tumor biology, suggesting that IGF2BP1 may serve as a favorable prognostic biomarker and therapeutic target. Further mechanistic and population-based studies are warranted to clarify its dualistic role in endometrial tumorigenesis.

## Introduction

Endometrial cancer (EC) is the most common gynecologic malignancy in developed countries. Although the incidence of EC in Asian populations remains lower than that observed in Western nations, it has shown a steady upward trajectory over recent decades. This trend is largely attributed to lifestyle westernization and population aging^1^. Asian women exhibit distinct epidemiological and clinical characteristics, including differences in molecular subtypes, exposure to risk factors, and treatment outcomes when compared to their Western counterparts^2^. Notably, despite a generally lower average body mass index (BMI), Asian populations demonstrate a disproportionately high prevalence of metabolic syndrome, which may drive tumorigenesis through unique metabolic and inflammatory mechanisms^3^. Furthermore, emerging evidence suggests that Asian patients may respond differently to treatment and exhibit distinct side-effect profiles, likely due to underlying pharmacogenetic and biological differences^4^. These observations underscore the need for region-specific epidemiological research and risk-stratification frameworks to guide personalized EC prevention and care strategies in Asian populations.

In addition to epidemiological disparities, genetic susceptibility patterns among Asian women with EC differ from those of Western populations. A recent large-scale analysis revealed that approximately 69.6% of Asian patients with EC harbor actionable genetic alterations, with frequent mutations in DNA repair-related genes such as *AKT1, MET, PMS2, PIK3R1*, and *CTCF*, pointing to new opportunities for precision oncology in this population^5^. Furthermore, several germline and somatic variants have been linked to increased EC risk in specific subgroups, including BCL2 gene polymorphisms in Chinese women^6^, XRCC1 SNPs in postmenopausal Japanese women^7^, and CYP19A1 haplotypes involved in estrogen metabolism^8^. The Pro allele at codon 72 of TP53 has also been associated with increased EC risk among Asians^9^. These findings emphasize the importance of incorporating molecular profiling into clinical protocols to improve risk prediction and therapeutic decision-making tailored to Asian populations.

Among potential molecular regulators of EC, insulin-like Growth Factor 2 mRNA-binding protein 1 (IGF2BP1) has emerged as a notable oncofetal RNA-binding protein. It enhances cancer progression by stabilizing oncogenic mRNAs via N6-methyladenosine (m^6^A)-dependent mechanisms. IGF2BP1's oncogenic roles have been well documented in colorectal, pancreatic, and hematologic malignancies, where it regulates cell cycle progression, metastasis, and therapeutic resistance^10^-^14^. In colorectal cancer, for example, IGF2BP1 promotes G1/S transition by stabilizing E2F1 transcripts and modulates tumor-derived extracellular vesicle content, contributing to treatment resistance. In leukemia, it maintains stemness and self-renewal in leukemia-initiating cells. These findings underscore its broader relevance as a pan-cancer biomarker and therapeutic target.

Caspase-3, a key executioner of apoptosis, is activated by upstream caspases and induces chromatin condensation and DNA fragmentation. In endometrial tissue, it functions as a critical switch from autophagy to apoptosis^15^. Ki-67 is a well-established marker of cellular proliferation and reflects changes in endometrial growth activity, making it a reliable indicator of proliferative potential and risk^16^. CD31 is an endothelial marker commonly used to assess microvessel density and angiogenesis, reflecting tumor vascular activity and aggressiveness in endometrial cancer^17^. Epithelial-mesenchymal transition (EMT) is a key biological process that enables epithelial tumor cells to lose cell-cell adhesion and polarity while acquiring mesenchymal properties, including enhanced motility and invasiveness. In uterine carcinosarcoma, a highly aggressive subtype of endometrial cancer, EMT facilitates the dedifferentiation of epithelial carcinoma cells into sarcomatous components, driving tumor heterogeneity, invasion, and progression^18^. This process is mediated by the downregulation of epithelial markers such as E-cadherin and the upregulation of mesenchymal markers such as N-cadherin and vimentin, leading to structural and functional changes that promote metastasis and therapeutic resistance^18^. AKT, a central serine/threonine kinase in the PI3K/AKT/mTOR signaling pathway, regulates essential processes such as cell survival, proliferation, metabolism, migration, angiogenesis, and the inhibition of apoptosis by phosphorylating downstream effectors such as BAD and Caspase-9^15^. Dysregulation of this pathway, often through PTEN loss or activating mutations, drives uncontrolled growth and therapeutic resistance in multiple cancers, including endometrial cancer^15^. ERK, a core component of the MAPK/ERK signaling pathway, mediates cellular responses to growth factors and stress stimuli, controlling proliferation, differentiation, survival, and motility^15^. In cancer, persistent ERK activation enhances transcriptional programs that promote cell cycle progression, survival, epithelial-mesenchymal transition, and resistance to therapy^15^. STAT3 plays a critical oncogenic role by maintaining persistent activation, often through Tyr705 phosphorylation, which upregulates anti-apoptotic genes such as Bcl-xL, survivin, and Mcl-1, thereby promoting tumor cell survival, proliferation, and therapeutic resistance^19^. In contrast, AMPK, a central energy sensor, exhibits a dual role in tumor biology. While traditionally recognized for suppressing tumor growth by maintaining energy homeostasis and inhibiting anabolic pathways, AMPK can also promote tumor progression under certain conditions. Recent evidence indicates that AMPK phosphorylation of INF2 facilitates mitochondrial fission and supports endometrial cancer cell proliferation, underscoring its context-dependent, tumor-promoting effects^20^.

In EC, IGF2BP1 is frequently overexpressed and has been linked to aggressive clinicopathologic features and poor prognosis^21^-^23^. Mechanistically, it promotes tumor EMT, and invasion by stabilizing oncogenic transcripts such as MYC and HMGA2, as well as modulating non-coding RNA networks (e.g., the linc01194-SOX2 axis) and PEG10 expression. However, most existing studies are limited to non-Asian populations, and the prognostic significance of IGF2BP1 in Asian EC cohorts remains poorly defined.

This study aims to address this gap by investigating the clinicopathological relevance and prognostic implications of IGF2BP1 expression in EC, with a specific focus on Asian patients. We aim to (1) evaluate its association with tumor grade, stage, and biomarker profiles; and (2) determine whether IGF2BP1 expression stratifies overall survival outcomes using Kaplan-Meier analysis. This work seeks to clarify IGF2BP1's potential role as a prognostic biomarker and therapeutic target in EC within the context of personalized cancer care.

## Materials and Methods

This study analyzed 75 tissue samples obtained from patients diagnosed with endometrial carcinoma between 2000 and 2014. All included patients were treatment-naïve at the time of initial tumor resection and had not received any form of neoadjuvant therapy, such as chemotherapy or radiotherapy, prior to surgery. Postoperative adjuvant treatments, including chemotherapy, radiotherapy, or combined modalities, were administered as indicated based on the pathological characteristics of each tumor specimen. This design ensured that the immunohistochemical and molecular analyses reflected the intrinsic biological profile of untreated tumor tissues, minimizing the potential confounding effects of preoperative therapies. Clinical and demographic data—such as sex, age, tumor size, histological grade, disease stage, and survival outcomes—were also collected. Ethical approval for this research was granted by the Cardinal Tien Hospital Ethics Committee (Approval No. CTH-106-2-5-042) on June 27, 2018.

For each specimen, a 2 mm tissue core was extracted and incorporated into a tissue microarray. The cores were paraffin-embedded, sectioned at 0.3 µm thickness, and mounted onto glass slides for immunohistochemical (IHC) evaluation. Prior to staining, slides were incubated at 65°C for 1 h to optimize tissue adherence. Deparaffinization and rehydration were achieved through sequential washes in xylene (2 × 10 min), absolute ethanol (5 min), graded ethanol series (95% and 75%, 5 min each), and a final distilled water rinse (10 min). Automated IHC staining was conducted using the Ventana BenchMark XT platform (Ventana Medical Systems, Tucson, AZ, USA).

**Figure 1:**
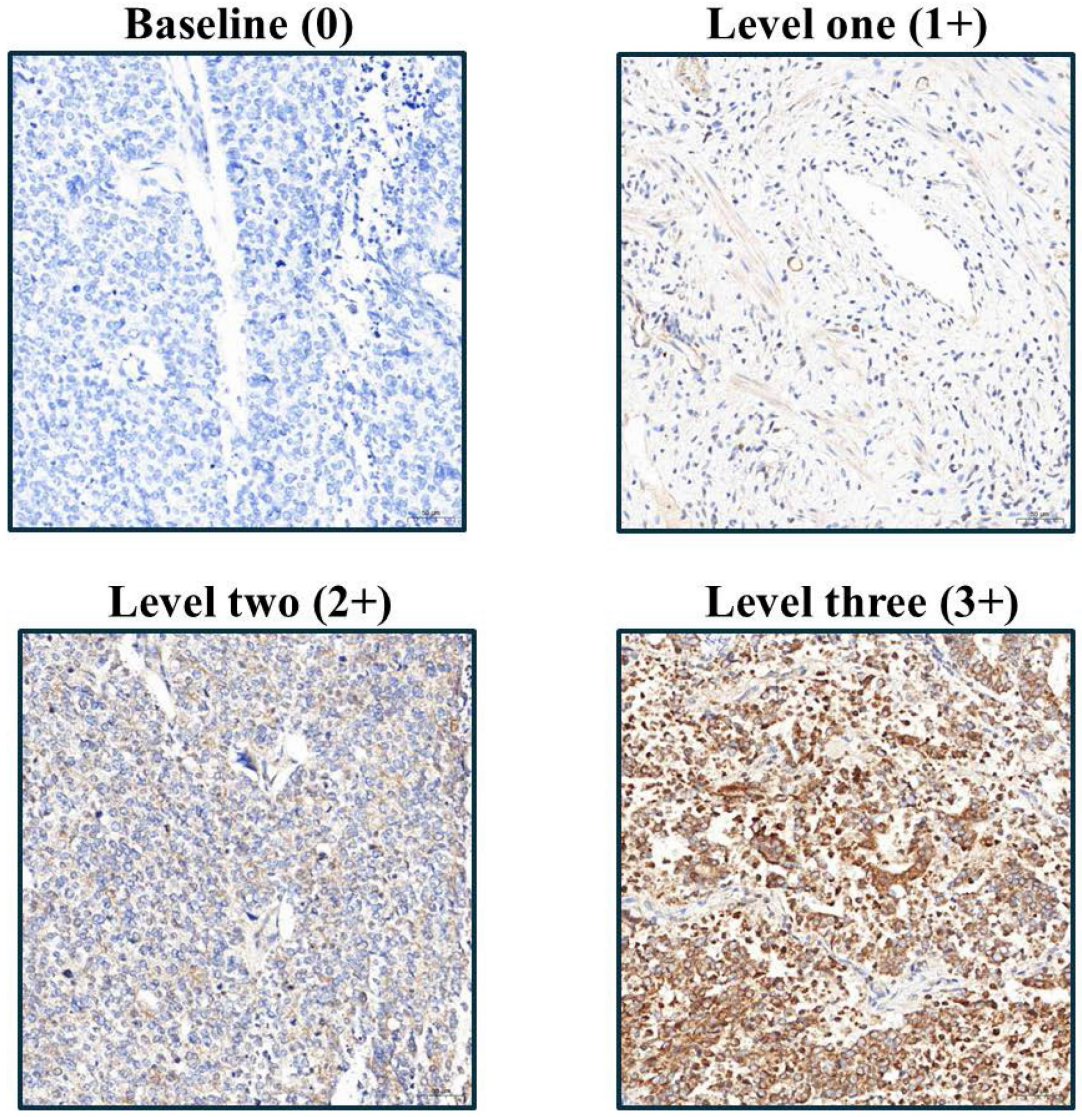
Immunohistochemical analysis of IGF2BP1 expression in endometrial cancer tissues, illustrating varying staining intensities corresponding to semi-quantitative scores from 1 to 3.

Following rehydration, tissue sections were rinsed with phosphate-buffered saline and then underwent EDTA-based antigen retrieval (24 min, conditions optimized per antibody). The following primary antibodies were used in this study: IGF2BP1 (Abcam, UK, ab82968), Caspase-3 (Cell Signaling, USA, 9664), Ki67 (BioLegend, USA, 350503), CD31 (Abbiotec, USA, 250590), E-cadherin (Abcam, UK, ab40772), N-cadherin (Abcam, UK, ab76011), Fibronectin (Santa Cruz, USA, SC-8422), pAkt (GeneTex, USA, GTX11901), pErk (R&D Systems, USA, AF1018), pStat3 (Abcam, UK, ab76315), and pAMPK (Cell Signaling, USA, 2535). These antibodies were selected based on their established specificity and were applied at optimized dilutions ranging from 1:50 to 1:500 according to manufacturer guidelines and experimental validation at 37°C for 1 h. Protein detection employed DAB chromogen with hematoxylin counterstaining, with all slides evaluated by Dr. Yen-Lin Chen (Tri-Service General Hospital Pathology) using a semi-quantitative H-score system: staining intensity (1-3 scale; Figure 1) multiplied by the ImageJ-quantified (v1.54f, NIH) positive area percentage, providing standardized expression analysis across apoptosis markers, proliferation indices, angiogenesis indicators, EMT regulators, and key phosphorylation targets.

To determine the optimal cutoff point for IGF2BP1 expression, a receiver operating characteristic (ROC) curve analysis was performed. The Youden Index was calculated for each possible H-score to evaluate its predictive performance for overall survival. An H-score of 10 demonstrated the best balance between sensitivity and specificity and was therefore selected as the threshold to categorize patients into low- and high-expression groups for subsequent analyses.

Based on an H-score threshold of 10, IGF2BP1 expression levels were classified into high- and low-expression groups. The distribution of continuous variables was examined using the Kolmogorov-Smirnov test, which indicated non-normality across all variables. Consequently, continuous data were summarized as medians with interquartile ranges and compared between groups using the Mann-Whitney U test. Categorical variables were reported as frequencies and percentages, and differences between groups were assessed using either the chi-square test or Fisher's exact test, depending on sample size. To explore factors associated with IGF2BP1 expression and clinical outcomes, univariate logistic regression analyses were initially performed. Variables found to be statistically significant were subsequently entered into a multivariate logistic regression model to identify independent prognostic indicators, with results reported as odds ratios (ORs) and 95% confidence intervals (CIs). Kaplan-Meier survival analysis was employed to compare outcomes between expression groups. All statistical procedures were conducted using SPSS version 26.0 (IBM Corp., Armonk, NY, USA), with a two-tailed p-value of less than 0.05 considered indicative of statistical significance.

## Results

A total of 75 patients with EC were included in the analysis, of whom 7 (9.3%) were classified as having low IGF2BP1 expression and 68 (90.7%) as having high expression, based on an H-score cutoff of 10. As shown in Table 1, no statistically significant differences were observed between the two groups in terms of age, tumor grade, size, or FIGO stage.

The median age was 54.0 years (IQR: 51.0-57.0) in the low-expression group and 56.0 years (IQR: 50.0-60.0) in the high-expression group (p = 0.848). Tumor grading did not differ significantly (p = 0.222), with well-differentiated tumors observed in 14.3% vs. 25.0%, moderately differentiated in 42.9% vs. 58.8%, and poorly differentiated in 42.9% vs. 16.2% of the low- and high-expression groups, respectively. The median tumor size was 1.5 cm (IQR: 0.8-6.0) in the low-expression group and 3.3 cm (IQR: 1.7-5.0) in the high-expression group (p = 0.372). FIGO stage distribution showed a predominance of Stage I disease in both groups (71.4% vs. 82.4%), with no significant difference in stage classification overall (p = 0.220).

Tumor biomarker expression levels were compared between low and high IGF2BP1 expression groups, as summarized in Table 2. Among the biomarkers analyzed, caspase-3 and phosphorylated Akt (pAkt) showed statistically significant differences between the two groups.

Median caspase-3 levels were significantly lower in the low IGF2BP1 expression group compared to the high-expression group (5.91 [IQR: 1.9-6.0] vs. 7.6 [IQR: 5.3-13.4], p = 0.019). Similarly, pAkt expression was markedly lower in the low-expression group (1.2 [IQR: 0.9-2.4]) than in the high-expression group (3.2 [IQR: 1.8-6.6], p = 0.035). Although fibronectin expression tended to be higher in the low-expression group (16.9 [IQR: 13.2-91.1]) than in the high-expression group (5.8 [IQR: 1.9-31.7]), the difference did not reach statistical significance (p = 0.080).

No significant differences were found for Ki67, CD31, E-cadherin, N-cadherin, pErk, pStat3, or pAMPK expression between the two groups (all p > 0.05).

To explore clinicopathological and molecular factors associated with high IGF2BP1 expression in EC, we performed both univariable and multivariable logistic regression analyses, as summarized in Table 3.

In the univariable analysis, caspase-3 expression was significantly associated with high IGF2BP1 expression, with an OR of 1.47 (95% CI: 1.01-2.12; p = 0.042), indicating that each unit increase in caspase-3 was associated with a 47% increase in the odds of high IGF2BP1 expression. Additionally, pStat3 expression showed a significant inverse association (OR = 0.66, 95% CI: 0.45-0.96; p = 0.031), suggesting that higher levels of phosphorylated signal transducer and activator of transcription 3 were associated with lower odds of IGF2BP1 overexpression.

Other factors, including age (OR = 1.01, p = 0.816), tumor size (OR = 1.13, p = 0.466), tumor grade (moderately differentiated: OR = 0.78, p = 0.838; poorly differentiated: OR = 0.22, p = 0.208), and FIGO stage (stage III: OR = 0.27, p = 0.161) were not significantly associated with IGF2BP1 expression. Among additional tumor biomarkers evaluated, none reached statistical significance, though pErk (OR = 0.96, p = 0.068) approached borderline significance.

Subsequently, variables with statistical significance in the univariable analysis (caspase-3 and pStat3) were included in the multivariable logistic regression model. In this adjusted model, both markers remained independently associated with high IGF2BP1 expression. Caspase-3 retained a significant positive association (adjusted OR = 1.88; 95% CI: 1.00-3.29; p = 0.026), while pStat3 maintained a significant inverse association (adjusted OR = 0.45; 95% CI: 0.20-0.99; p = 0.047).

These results suggest that elevated apoptotic activity, reflected by increased caspase-3, and reduced activation of signal transducer and activator of transcription 3 (STAT3) signaling may independently correlate with IGF2BP1 overexpression in EC tissue. The findings support the potential mechanistic role of IGF2BP1 in modulating apoptotic and inflammatory signaling pathways.

Patients were categorized into high and low IGF2BP1 expression groups using a cutoff score of 10. The Kaplan-Meier survival analysis revealed a clear separation between the two groups over time (log-rank p = 0.029). The high-expression group (IGF2BP1 > 10), depicted in dark blue, demonstrated a more favorable overall survival, maintaining a survival probability above 85% throughout the follow-up period. In contrast, the low-expression group (IGF2BP1 ≤ 10), shown in light blue, exhibited a markedly poorer prognosis, with survival probability declining to below 40% by year 4.

Although the plot suggests a trend toward poorer survival among patients with low IGF2BP1 expression, statistical significance should be verified using a log-rank test. Confidence intervals are shown as shaded areas along the survival curves.

## Discussion

In summary, our study provides novel insights into the clinicopathological and prognostic significance of IGF2BP1 in EC, particularly in an Asian patient population. While IGF2BP1 has been broadly implicated in multiple malignancies, its role in EC remains under characterized. Through comprehensive analysis integrating IHC profiling, biomarker correlation, and survival modeling, we identified a distinct association between IGF2BP1 expression and key oncogenic markers, including caspase-3 and pStat3, suggesting its involvement in apoptosis regulation and inflammatory signaling. Importantly, Kaplan-Meier survival analysis revealed that patients with differential IGF2BP1 expression exhibited markedly divergent survival trajectories, indicating its potential utility as a prognostic biomarker. These findings support the hypothesis that IGF2BP1 may serve as a critical molecular indicator in EC pathogenesis and patient stratification, warranting further investigation in larger, multi-ethnic cohorts and prospective studies.

Previous studies have reported that IGF2BP1 is frequently overexpressed in EC and is associated with aggressive clinicopathological features and poor patient prognosis, largely through its stabilization of oncogenic mRNAs such as MYC and HMGA2, and regulation of lncRNA-related pathways like the linc01194-SOX2 axis^21^-^23^. However, these findings have predominantly been derived from Western cohorts, and data on IGF2BP1 expression and its prognostic implications in Asian populations with EC remain scarce.

In contrast to earlier reports emphasizing the oncogenic role of IGF2BP1, our study uniquely focuses on an Asian cohort and reveals a divergent survival pattern associated with IGF2BP1 expression. Compared to previously published literature^23^, our study demonstrates distinct differences and innovations in patient cohort, analytical approach, and clinical implications. The prior study enrolled 96 patients with EC from Beijing and Nanjing, China, and focused on elucidating the molecular mechanism by which IGF2BP1 stabilizes PEG10 mRNA to promote oncogenesis, primarily using *in vitro* and *in vivo* models. In contrast, our study involved 75 patients from Taiwan and explicitly excluded individuals who had undergone chemotherapy or radiotherapy, allowing for a more accurate assessment of untreated tumor progression. Additionally, while earlier studies relied on median expression levels for group stratification, we employed receiver operating characteristic curve analysis to determine the optimal prognostic cutoff (cutoff = 10) and validated survival differences using Kaplan-Meier and log-rank tests, revealing significantly better survival in the high-expression group (p = 0.029). Beyond this, we also investigated correlations between IGF2BP1 and various IHC biomarkers, such as caspase-3 and pSTAT3, and identified associations with apoptotic and inflammatory signaling. These findings underscore the potential of IGF2BP1 as a prognostic and biological marker in Taiwanese patients with EC, addressing limitations in prior studies that predominantly focused on Western or mainland Chinese populations. This discrepancy may reflect underlying differences in tumor biology, genetic background, treatment response, or IGF2BP1-associated signaling mechanisms between Asian and Western populations. Additionally, our biomarker correlation analyses provide new insight into IGF2BP1's potential interaction with apoptotic and inflammatory regulators such as caspase-3 and pStat3, highlighting molecular axes that have not been previously reported in EC.

Caspase-3 is a key executioner protease in the intrinsic apoptosis pathway and has long been recognized for its tumor-suppressive role through the induction of programmed cell death. Its activation leads to the cleavage of various cellular substrates, culminating in the irreversible dismantling of the cell. In multiple cancer types, including breast and colorectal cancers, increased caspase-3 activity has been associated with reduced tumor proliferation and improved therapeutic responses^24^, ^25^. Moreover, restoration of caspase-3-mediated apoptosis has been proposed as a promising strategy for overcoming resistance to chemotherapy and radiotherapy^15^, ^26^.

In our study, we observed that high IGF2BP1 expression was independently associated with elevated caspase-3 levels, suggesting a potential functional interplay between these two molecules in EC. Interestingly, contrary to previous studies linking IGF2BP1 overexpression to tumor aggressiveness^22^, ^23^, our findings revealed that patients with high IGF2BP1 expression—and concomitantly elevated caspase-3—exhibited better overall survival. This unexpected association implies that in specific molecular contexts, such as within Asian populations with EC, IGF2BP1 overexpression may trigger compensatory apoptotic mechanisms that attenuate tumor progression. The multivariable analysis further supported caspase-3 as an independent factor associated with IGF2BP1 status, reinforcing the notion that increased apoptotic potential may underlie the favorable prognosis observed in this subgroup^27^.

These findings provide a new perspective on the dualistic nature of IGF2BP1 in cancer biology and highlight the relevance of caspase-3 as a downstream effector potentially mitigating the oncogenic effects of RNA-binding proteins.

STAT3 is a well-established oncogenic transcription factor that promotes tumorigenesis by regulating genes involved in cell proliferation, survival, immune suppression, and angiogenesis. Aberrant and persistent activation of phosphorylated STAT3 (pSTAT3) has been documented across a wide range of malignancies and is commonly associated with poor prognosis and treatment resistance^28^, ^29^. For example, in hepatocellular carcinoma and colorectal cancer, STAT3 activation facilitates tumor progression via IL-6/JAK/STAT3 signaling, driving EMT and metastatic potential^30^, ^31^. In addition, STAT3 contributes to immune evasion by upregulating PD-L1 and suppressing cytotoxic T-cell infiltration^32^, ^33^.

In contrast to these widely reported pro-tumorigenic effects, our study identified a significant inverse association between IGF2BP1 expression and pSTAT3 levels in EC tissues. While IGF2BP1 is generally characterized as an oncogenic RNA-binding protein that stabilizes m^6^A-modified transcripts to support tumor growth, its high expression in our cohort was independently associated with reduced levels of pSTAT3 and, notably, with improved overall survival. This finding deviates from the typical paradigm observed in other cancers, suggesting that IGF2BP1 may exert context-dependent effects on STAT3 signaling in EC.

One possible explanation is that IGF2BP1 may stabilize negative regulators of STAT3 activation or modulate upstream signaling events that suppress STAT3 phosphorylation in a tissue-specific manner. Alternatively, the observed downregulation of pSTAT3 in high-IGF2BP1 tumors may reflect compensatory mechanisms or shifts in immune signaling dynamics unique to the endometrial tumor microenvironment. Regardless of the mechanism, our findings highlight the complexity of the IGF2BP1-STAT3 axis and underscore the need for cancer-type-specific investigations into their functional interplay.

Collectively, this unexpected inverse relationship between IGF2BP1 and pSTAT3 provides new insights into EC biology and suggests that IGF2BP1 may modulate tumor progression not solely through classical oncogenic mRNA stabilization, but also via suppression of key inflammatory and pro-survival signaling pathways such as STAT3^28^-^33^.

Emerging evidence indicates that IGF2BP1 plays a critical role in regulating PKM2 expression and activity across different disease contexts. In bladder cancer, IGF2BP1 directly stabilizes PKM2 mRNA, while circFAM13B competes for IGF2BP1 binding, leading to PKM2 destabilization, reduced glycolysis, and improved sensitivity to immunotherapy^34^. In endometriosis models, IGF2BP1 knockdown decreased the expression of key glycolytic enzymes, including PKM2 and HK2, and reduced glucose uptake, highlighting IGF2BP1's role in supporting metabolic reprogramming through PKM2 regulation^35^. Although the study on pulmonary fibrosis did not directly evaluate PKM2, it demonstrated that IGF2BP1 broadly modulates mRNA stability through m6A-dependent mechanisms, suggesting a potential pathway by which IGF2BP1 could influence PKM2 stability in other tissues^36^. Collectively, these findings suggest that IGF2BP1 may orchestrate PKM2-driven metabolic pathways through both direct mRNA stabilization and indirect regulatory mechanisms. While this interaction remains unexplored in endometrial cancer, the mechanistic insights from other systems provide a strong rationale for investigating whether IGF2BP1 similarly regulates PKM2 expression and contributes to metabolic reprogramming and tumor progression in this context.

Taken together, our findings challenge the prevailing view of IGF2BP1 as a uniformly pro-tumorigenic factor, suggesting that its role in EC may be more nuanced and context-dependent, particularly within Asian populations. The independent associations of high IGF2BP1 expression with elevated caspase-3 and reduced pSTAT3 levels highlight its potential involvement in modulating both apoptotic and inflammatory pathways, which may contribute to favorable clinical outcomes. These results not only expand our understanding of IGF2BP1-mediated signaling in EC but also underscore the importance of considering molecular interactions and ethnic-specific tumor biology in biomarker evaluation. Further mechanistic studies and large-scale validation across diverse cohorts are warranted to elucidate the dualistic role of IGF2BP1 and explore its clinical utility as a prognostic indicator and therapeutic target in EC.

## Limitations

Several limitations should be acknowledged. First, the sample size was relatively small and obtained from a single institution, which may limit the generalizability of our findings. Second, the observational design precludes establishing definitive causal relationships between IGF2BP1 expression and survival outcomes. Third, although immunohistochemistry provided valuable protein-level insights, functional and mechanistic experiments, such as knockdown or overexpression models, were not performed and will be necessary to clarify the underlying biological pathways. Fourth, this study exclusively focused on an Asian population; thus, validation in larger, multi-ethnic cohorts is needed to determine whether IGF2BP1 serves as a universal or population-specific biomarker. Finally, there was an imbalance in expression group sizes, with far fewer patients in the low IGF2BP1 expression group (N=7) compared with the high-expression group (N=68), potentially limiting statistical power. Although this distribution aligns with patterns observed in the TCGA UCEC dataset, larger and more balanced cohorts will be essential to confirm the robustness and generalizability of our findings.

## Conclusion

Our study highlights the prognostic significance of IGF2BP1 in EC among Asian patients, revealing a paradoxical association between high IGF2BP1 expression and improved survival. This effect may be mediated by increased caspase-3 and reduced pSTAT3 activity, suggesting a context-specific role in apoptosis and inflammation. These findings support reconsidering IGF2BP1 as a potential biomarker for tailored risk assessment and therapy across diverse populations.

## Figures and Tables

**Figure 2:**
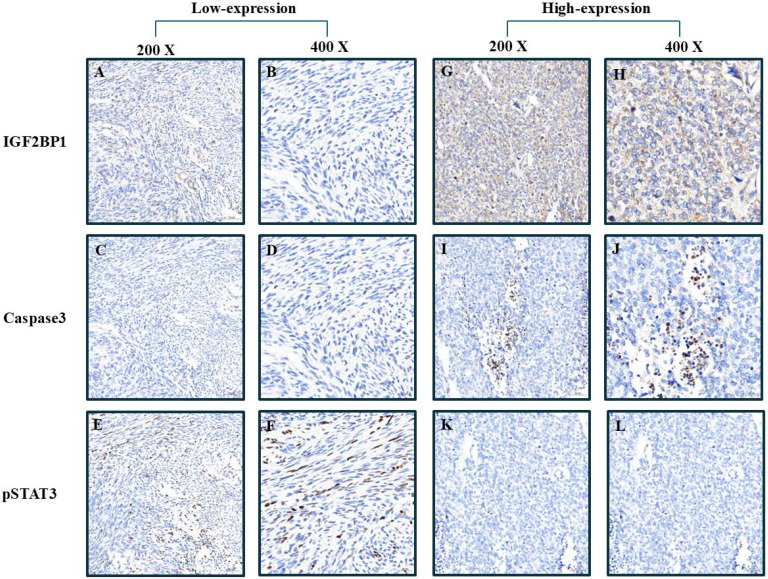
Comparative visualization of biomarker distribution in tumor samples stratified by IGF2BP1 expression level. Figures A-C represent low magnification views (200×), while D-E display high magnification details (400×), highlighting differential staining patterns.

**Figure 3:**
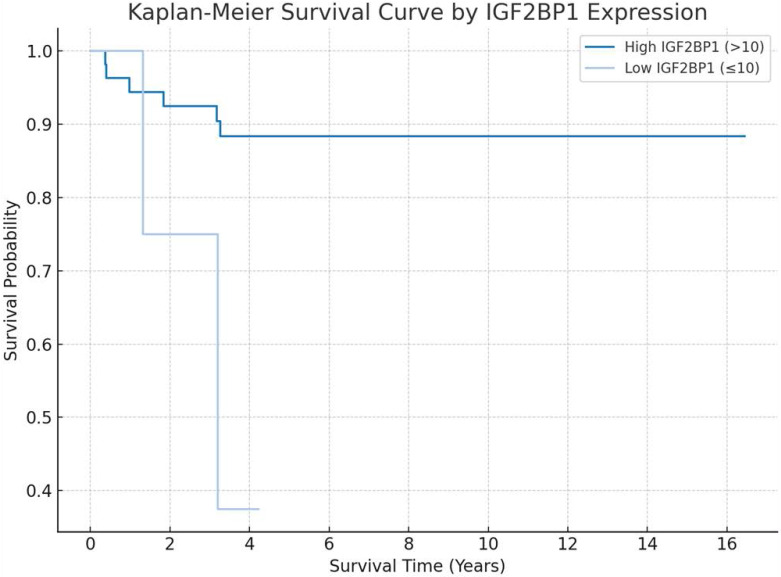
Kaplan-Meier survival analysis demonstrating the prognostic significance of IGF2BP1 expression, with distinct overall survival trends observed between low- and high-expression groups.

**Table 1:** Comparison of Clinicopathological Characteristics Between IGF2BP1 Expression Groups

	Low expression of IGF2BP1 (N=7)	High expression of IGF2BP1 N=68)	Total (N=75)	p-value
Age	54.0 (51.0, 57.0)	56.0 (50.0, 60.0)	56.0 (50, 60)	0.848
Grading				0.222
Well	1 (14.3%)	17 (25.0%)	18 (24.0%)	
Moderate	3 (42.9%)	40 (58.8%)	43 (57.3%)	
Poorly	3 (42.9%)	11 (16.2%)	14 (18.7%)	
Size	1.5 (0.8, 6.0)	3.3 (1.7, 5.0)	3.0 (1.5, 5.0)	0.372
Stage				0.220
I	5 (71.4%)	56 (82.4%)	61 (81.3%)	
II	0 (0.0%)	6 (8.8%)	6 (8.0%)	
III	2 (28.6%)	6 (8.8%)	8 (10.7%)	

**Table 2:** Comparison of Tumor Biomarker Expression Between Low- and High-IGF2BP1 Expression Groups in Endometrial Cancer

	Low expression of IGF2BP1	High expression of IGF2BP1	Total	p-value
Caspase-3	5.91 (1.9, 6.0)	7.6 (5.3, 13.4)	7.1 (5.2, 12.8)	0.019*
Ki67	3.0 (1.9, 5.4)	5.3 (1.3, 18.0)	5.2 (1.3, 16.1)	0.209
CD31	7.7 (4.1, 13.8)	6.8 (5.1, 9.5)	6.9(5.1, 96)	0.985
E-cadherin	102.1 (100.5, 117.2)	109.5 (104.4, 116.0)	109.4 (103.5, 116.1)	0.209
N-cadherin	5.8 (1.3, 34.1)	8.3 (2.8, 34.5)	7.6 (2.5, 34.1)	0.423
Fibronectin	16.9 (13.2, 91.1)	5.8 (1.9, 31.7)	8.9 (2.1, 31.9)	0.080
pAkt	1.2 (0.9, 2.4)	3.2 (1.8, 6.6)	3.0 (1.6, 6.5)	0.035*
pErk	0.6 (0.0, 31.8)	0.4 (1.8, 6.6)	0.4 (0.1, 4.6)	0.978
pStat3	0.1 (0.1, 7.0)	0.1 (0.1, 0.4)	0.1 (0.1, 0.4)	0.560
pAMPK	14.9 (12.7, 16.8)	14.7 (11.6, 22.7)	14.8 (12.0, 22.6)	0.729

*P <0.05

**Table 3:** Univariable and Multivariable Logistic Regression Analysis of Factors Associated with High-IGF2BP1 Expression in Endometrial Cancer

	Univariable	p-value	Multivariable	p-value
Age	1.01 (0.93-1.10)	0.816		
Grading				
Well	Reference			
Moderate	0.78 (0.08-8.09)	0.838		
Poorly	0.22 (0.02-2.35)	0.208		
Size	1.13 (0.82-1.56)	0.466		
Stage				
I	Reference			
II	144238825.3 (0.00-)	0.999		
III	0.27 (0.04-1.69)	0.161		
Marker				
Caspase-3	1.47 (1.01-2.12)	0.042	1.88 (1.00-3.29)	0.026*
Ki67	1.09 (0.96-1.25)	0.186		
CD31	1.03 (0.90-1.18)	0.687		
E-cad	1.97 (0.95-1.20)	0.274		
N-cad	1.00 (0.97-1.04)	0.840		
Fibronectin	0.99 (0.97-1.01)	0.279		
pAkt	1.20 (0.85-1.67)	0.298		
pErk	0.96 (0.91-1.00)	0.068		
pStat3	0.66 (0.45-0.96)	0.031	0.45 (0.20-0.99)	0.047*
pAMPK	1.03 (0.93-1.13)	0.608		

*P <0.05
